# Transfusion-Free Survival Predicts Severe Retinopathy in Preterm Neonates

**DOI:** 10.3389/fped.2022.814194

**Published:** 2022-02-10

**Authors:** Luciana Teofili, Patrizia Papacci, Martina Bartolo, Anna Molisso, Nicoletta Orlando, Lucia Pane, Carmen Giannantonio, Francesca Serrao, Maria Bianchi, Caterina Giovanna Valentini, Claudio Pellegrino, Antonio Baldascino, Brigida Carducci, Domenico Lepore, Giovanni Vento

**Affiliations:** ^1^Divisione di Medicina Trasfusionale, Dipartimento di Diagnostica per Immagini, Radioterapia Oncologica, ed Ematologia, Fondazione Policlinico Universitario A. Gemelli Istituto di Ricerca e Cura a Carattere Scientifico, Rome, Italy; ^2^Sezione di Ematologia, Dipartimento di Scienze Radiologiche ed Ematologiche, Università Cattolica del Sacro Cuore, Rome, Italy; ^3^Divisione di Neonatologia, Dipartimento per le Scienze della Salute della Donna, del Bambino e di Sanità Pubblica, Fondazione Policlinico Universitario A. Gemelli, Istituto di Ricerca e Cura a Carattere Scientifico, Rome, Italy; ^4^Dipartimento di Scienze della Vita e Sanità Pubblica, Università Cattolica del Sacro Cuore, Rome, Italy; ^5^Divisione di Oculistica, Dipartimento di Scienze dell'Invecchiamento, Neurologiche, Ortopediche e della Testa-Collo, Fondazione Policlinico Universitario A. Gemelli Istituto di Ricerca e Cura a Carattere Scientifico, Rome, Italy; ^6^Divisione di Ostetricia e Patologia Ostetrica, Dipartimento per le Scienze della Salute della Donna, del Bambino e di Sanità Pubblica, Fondazione Policlinico Universitario A. Gemelli, Istituto di Ricerca e Cura a Carattere Scientifico, Rome, Italy; ^7^Dipartimento Testa-Collo e Organi di Senso, Università Cattolica del Sacro Cuore, Rome, Italy

**Keywords:** postmenstrual age, fetal hemoglobin, RBC transfusion, preterm birth, retinopathy

## Abstract

Repeated red blood cell (RBC) transfusions are thought to increase the risk for retinopathy of prematurity (ROP), likely due to a critical fetal hemoglobin (HbF) reduction. In this study, we investigated if the postmenstrual age (PMA) of neonates at transfusion influences the risk for ROP. We estimated the cumulative transfusion-free survival (TFS) in a series of 100 preterm neonates receiving one or more RBC units. TFS was calculated by censoring patients at first transfusion and expressing the time between birth and transfusion as either PMA or postnatal day. Then, we investigated if TFS predicted the occurrence of severe ROP, defined as ROP stage 3 or higher. We found that neonates with severe ROP displayed a significantly shorter TFS expressed according to their PMA (*p* = 0.001), with similar TFS according to postnatal days. At receiver operating characteristic (ROC) curve analysis, receiving an RBC unit before week 28 of PMA predicted severe ROP with a sensitivity of 64% and a specificity of 78%. In addition, receiving a second RBC unit before the PMA of 29 weeks predicted severe ROP with a sensitivity of 75% and a specificity of 69%. At multivariate analysis, PMA at the second transfusion was even more informative than at first transfusion and outperformed all other variables in predicting severe ROP, with an odds ratio of 4.554 (95% CI 1.332–15.573, *p* = 0.016). Since HbF decrease is greater after multiple RBC transfusions, it is conceivable that neonates receiving more than one unit before the PMA of 29 weeks may be exposed to a greater disturbance of retinal vascularization. Any strategy aimed at preventing the critical HbF decrease at this low age might potentially reduce the risk for severe ROP.

## Introduction

Extremely preterm birth is associated with a high risk for morbidity and mortality (1). Along with the decline of mortality, attention has been focused on long-term morbidities and related functional impairments,which lifelong affect the quality of life of “born too soon” neonates (1). Among them, the retinopathy of prematurity (ROP) still represents one of the major causes of blindness in childhood (2, 3). The pathogenesis of ROP relies on an initial phase of vessel growth inhibition, followed by hypoxia-induced neovascularization in the retina becoming metabolically active (4). Low gestational age (GA) and birth weight are the main risk factors for ROP, and in a fraction of cases, a genetic predisposition further increases this risk (4, 5). After the initial description of ROP, it has been rapidly recognized that excessive oxygen exposure promotes disease development (6). Despite current oxygen therapy protocols preventing hyperoxia through controlled supplementation, many preterm neonates still experience severe forms of ROP and frequently require appropriate treatments (2, 3). Among other risk factors, repeated red blood cell (RBC) transfusions are alleged to favor the ROP development. Apparently, the association between RBC transfusions and morbidity or mortality seems indisputable (7–9). In addition, there is evidence that RBC transfusions promote in preterm neonates the release of mediators of the inflammatory response and endothelial cell activation (10, 11). Overall, it is plausible that the detrimental effect of RBC transfusions may be driven by oxidative stress that cannot be managed by the immature antioxidant system of preterm neonates (12). Even so, preterm infants are among the most heavily transfused patients, with more critically ill neonates also receiving larger amounts of transfusions (13). In fact, these patients experience infections, hemorrhages, and surgical emergencies; they invariably develop a severe anemia consequent to the prematurity itself and the repeated blood sampling (13, 14).

**Figure 1:**
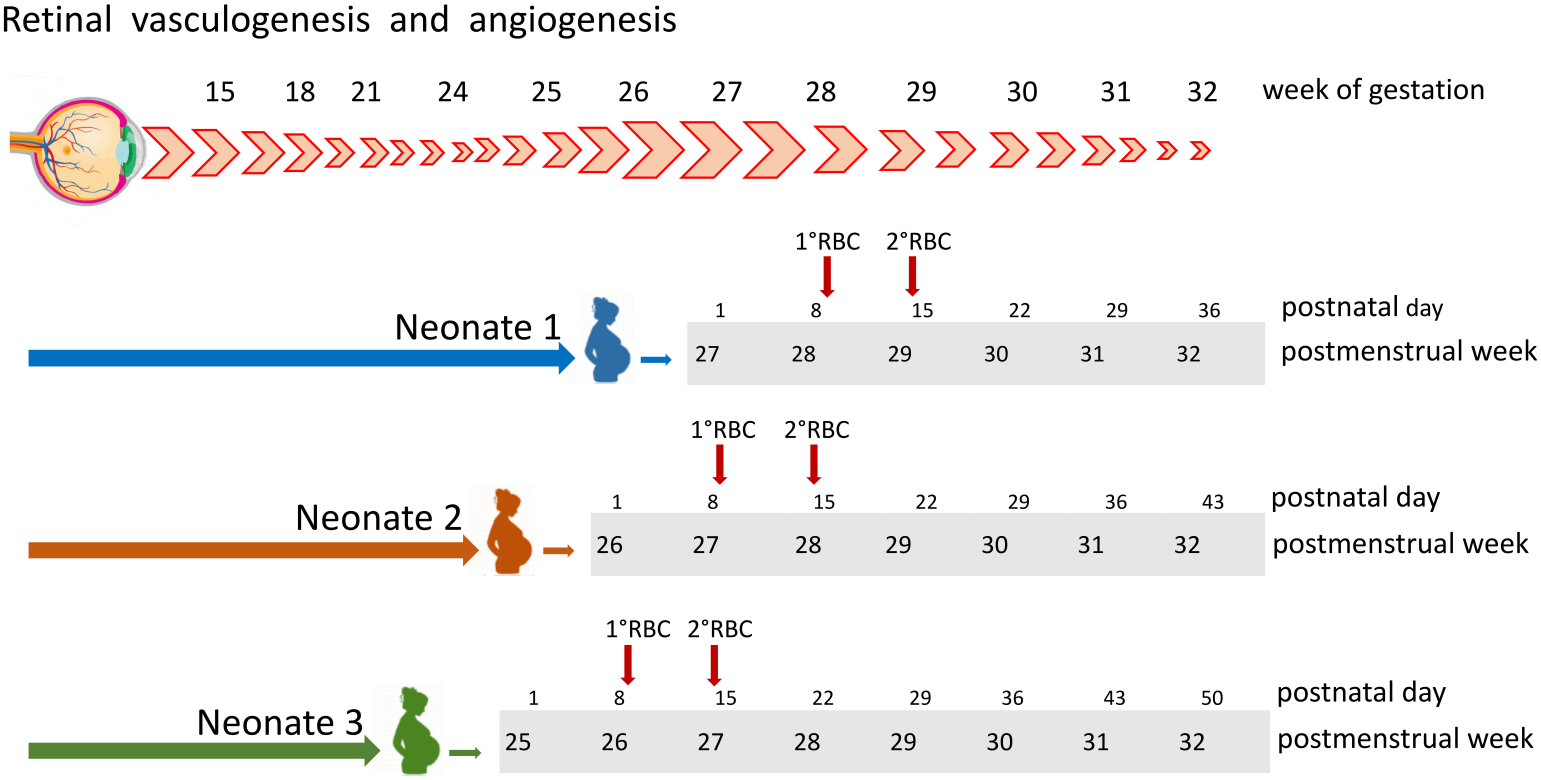
Retinal vessels development occurs through vasculogenesis and angiogenesis in a well-specified period of gestation, starting before week 15 and concluding around week 31. At the bottom are displayed the cases of three neonates, all receiving two RBC units at postnatal days 9 and 14 (red arrows). According to postmenstrual age, at the beginning of week 28, patient 1 had received no transfusion, patient 2 had received one single transfusion, and patient 3 was given two transfusions. RBC, red blood cell.

In contrast with previous studies, two large prospective trials and a recent meta-analysis comparing restrictive or liberal transfusion approaches in preterm neonates failed to confirm the negative effect of transfusions on neonate morbidity and mortality (15–17). This apparent discrepancy may be explained by hypothesizing that in a given patient the detrimental effect of transfusions may depend on the combination of multiple factors, including the number of transfused units, the interval between transfusions, and the postmenstrual age (PMA) of the recipient at various transfusion events. In fact, retinal vessel formation occurs in specific weeks of gestation (Figure 1) (18). Therefore, we hypothesized that transfusions given at an even slightly advanced stage of development might be less harmful than those given at lower age. This study aimed at estimating the association between ROP and age at transfusion, in order to assess if transfusions administered at lower PMA convey a higher risk for developing ROP. For this purpose, we calculated the cumulative transfusion-free survival (TFS) in a series of preterm neonates receiving one or more RBC transfusions. Then, we investigated if TFS was associated with the occurrence of severe ROP, defined as ROP stage 3 or higher.

## Materials and Methods

### Study Description

This is a retrospective study exploring the cumulative TFS in a series of preterm neonates born at GA < 30 weeks. The primary aim was to estimate the TFS in neonates with or without severe ROP, defined as a ROP at stage III or higher (19). Secondarily, we investigated if this parameter was associated with severe ROP.

### Study Population

**Figure 2:**
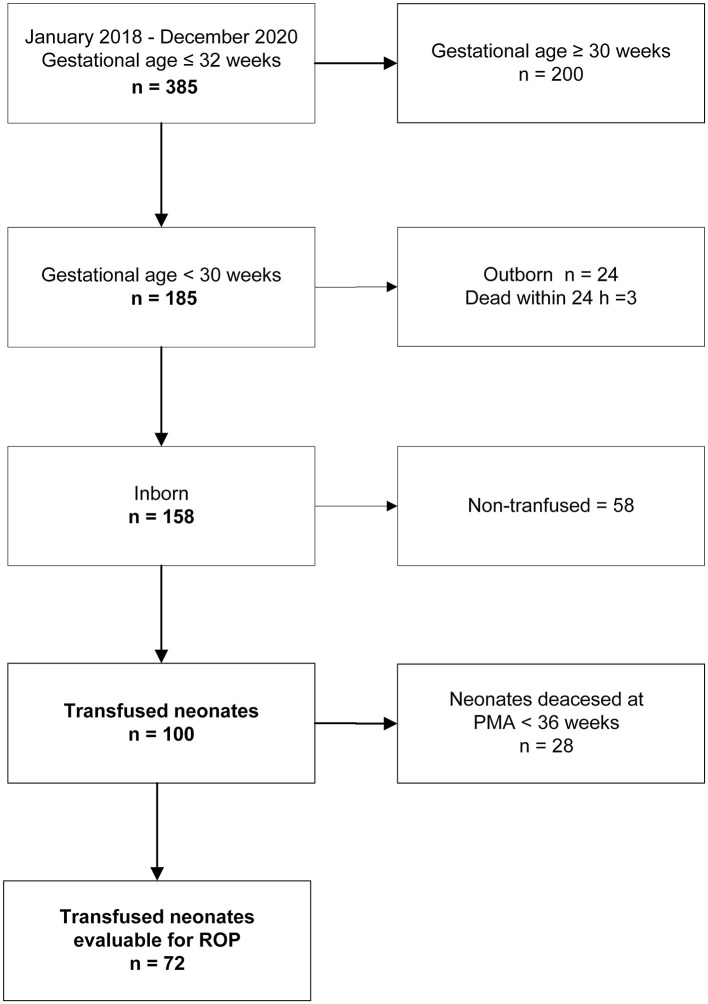
Flow diagram on the inclusion/exclusion criteria and final cohort of investigated patients. PMA, postmenstrual age; ROP, retinopathy of prematurity.

Clinical records of neonates with GA ≤ 32 weeks admitted to the Neonatal Intensive Care Unit (NICU) of Fondazione Policlinico A. Gemelli IRCCS between January 2018 and December 2020 were reviewed. Originally, the search was conducted to estimate the sample size of a prospective interventional study entitled “Umbilical or adult donor RBC to transfuse extremely low gestational age neonates. A randomized trial to assess the effect on ROP severity” (study ID 4364, ethics committee approval N 0027862/21; identifier ClinicalTrials.gov NCT05100212). From this initial population, we selected neonates born in the hospital at GA < 30 weeks who received at least one RBC unit (Figure 2). We decided to use the GA threshold of 30 weeks to include in the present study only patients with a reasonable risk for ROP.

### Study Definitions

Cumulative TFS was estimated censoring patients at the first RBC transfusion event. The age at transfusion was expressed in terms of PMA or postnatal day. This approach is simplified in Figure 1, illustrating retinal vessel development along gestation weeks and transfusion events in three different patients. According to the PMA of 28.0 weeks, patient 1 received no transfusion, patient 2 received one single transfusion, and patient 3 was given two transfusions. Conversely, expressing transfusion time as postnatal days, all 3 patients received 2 RBC transfusions in the first 15 days of life (day 9 and 14, Figure 1). Throughout the study period, ROP examinations were performed by the same experienced pediatric ophthalmologists (DL and AB), and diagnosis and staging of ROP were performed according to standardized criteria (19). Decisional algorithms for transfusing were the same over the study period and have been detailed elsewhere (20).

### Data Collection

Data were collected through i.t. databases in use at our hospital: Digistat (Ascom, Scandicci, Italy) for neonatal records and Emonet (version 0.91012000 1998–2020 Insiel Mercato GPI group, Trento, Italy) for transfusion data. The following variables were recorded: date of birth, GA at birth (week and days, expressed as the fraction of 1 week); birth weight (BW; g); BW centile; small for GA (SGA) status, and gender; Apgar index measured at 1 and 5 min; hematocrit (Htc) at birth; microbiologically documented infections; maximal stage and date of diagnosis of ROP (19); need for ROP treatment; occurrence and date of intraventricular hemorrhage (IVH) (21); occurrence of necrotizing enterocolitis (NEC) requiring surgery (22, 23); duration of ventilator support (invasive and non-invasive, days); O_2_ therapy (days); the occurrence of bronchopulmonary dysplasia (24); number and date of transfusions of RBC; platelet (PLT) and fresh frozen plasma (FFP) units; date of last follow up; and date of death.

### Statistical Analysis

Continuous variables were expressed as median [interquartile range (IQR)] and categorical variables as *n* (%). To compare continuous variables, we used the Mann–Whitney *U*-test or the Kruskal–Wallis test, as appropriate; for categorical variables, we used Fisher's exact test or the χ^2^ test, as appropriate. Cumulative TFS was evaluated using the Kaplan–Meier method, and comparison between curves was performed according to the log-rank test and expressed as hazard ratio (HR), with relative 95% CI. The combined effect of different variables on severe ROP was evaluated by multivariate logistic regression analysis (backward stepwise method) after centering collinear variables and including as covariates those with a significant effect (*p* < 0.05), on the outcome at univariate analysis. The test of Hosmer and Lemeshow was used to assess the quality of the models. For specific continuous variables, a receiver operating characteristic (ROC) curve analysis was performed in relation to the severe ROP outcome: the best cutoff identified according to sensitivity and specificity values was used as a categorical variable in the multivariate analysis. Results were expressed as odds ratio (OR) with relative 95% CI. Analyses were performed using the NCSS 10 (v 10.0.19 NCSS, LLC, Kaysville, UT, USA) and IBM SPSS Statistics for Windows (Version 27.0. IBM Corp. Released 2020, Armonk, NY, USA). The data supporting the findings of this study are available from the senior author upon reasonable request.

## Results

Among 385 preterm neonates initially evaluated, 185 infants had a GA at birth <30 weeks. We excluded 24 outborn neonates and 3 infants who deceased within 24 h. Of the remaining 158 patients, 100 received at least 1 RBC transfusion and were evaluable for the TFS: 72 of them reached the PMA of 36 weeks and were evaluable for ROP (Figure 2).

### Transfusion-Free Survival in the Study Population

**Figure 3:**
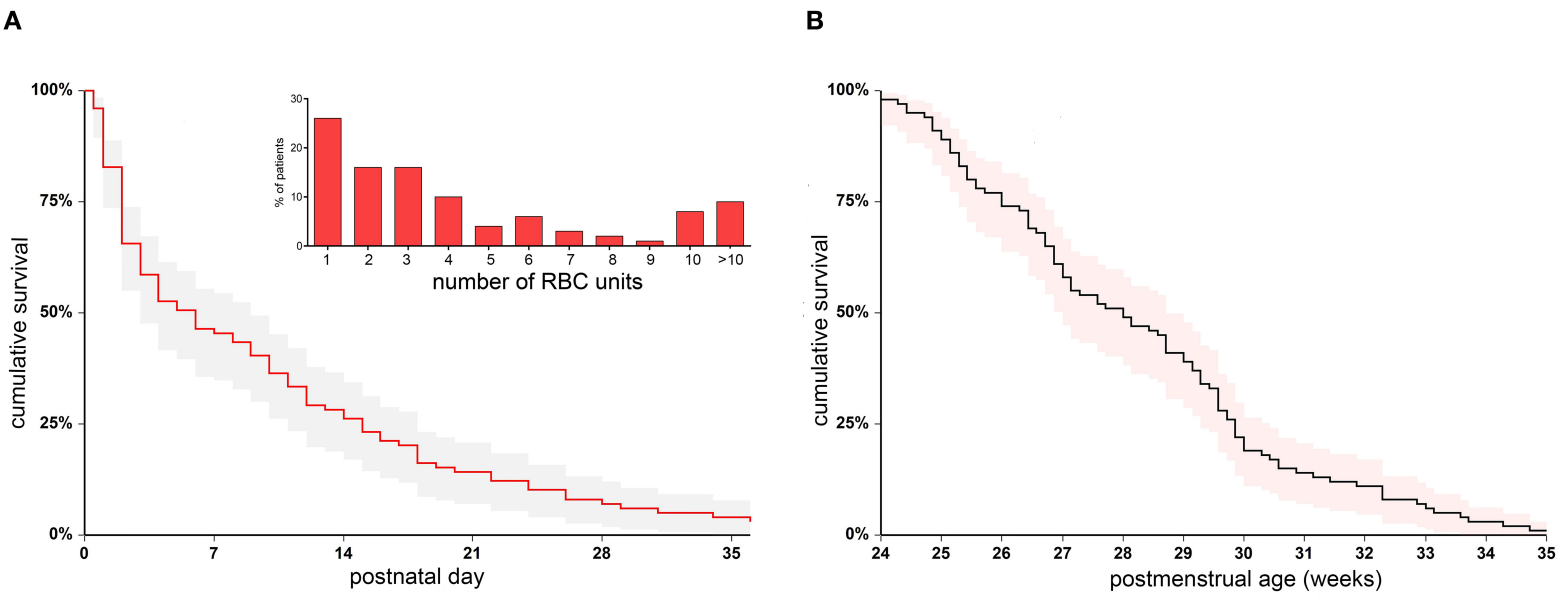
Cumulative transfusion-free survival in the initial cohort of 100 transfused patients. The survival was estimated censoring patients at first RBC transfusion event, expressed according to postnatal day **(A)** or postmenstrual weeks **(B)**. The distribution of RBC unit needs is also displayed (inset in **A**). RBC, red blood cell.

**Figure 4:**
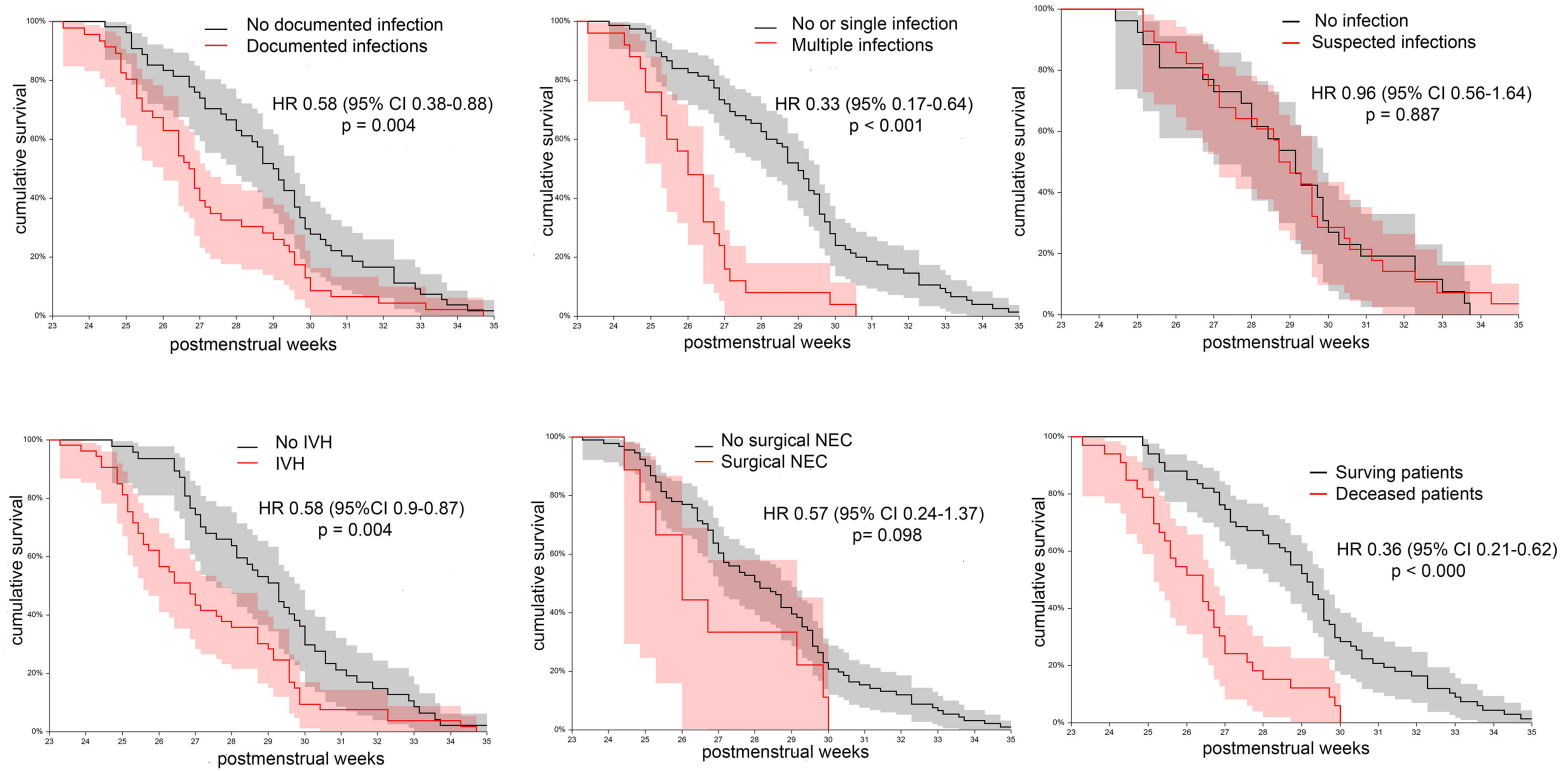
Cumulative transfusion-free survival in the initial cohort of 100 transfused patients. Patients were variably grouped according to different clinical outcomes: documented infections, multiple documented infections, suspected infectious episodes not followed by pathogen identification, intraventricular hemorrhage (IVH), necrotizing enterocolitis requiring surgery, and death.

Clinical characteristics of 100 neonates receiving transfusions are shown in Table 1. Median GA at birth was 27.0 weeks (25.0–28.3), and median BW was 795 g (666 – 1,067). The median follow-up was 80 days (32–111), and the median PMA at the last observation was 40.8 weeks (38.0–44.5). All patients received at least one RBC transfusion, and 23 of them were given also PLT units and 8 FFP units. In patients who received different blood products, the transfusion of RBC was antecedent or concomitant to the transfusion of other blood products in 88.4% of cases. Figure 3 shows patient distribution according to the RBC unit consumption and the cumulative TFS of the investigated patients. Censoring patients at postnatal day at transfusion, the median TFS was recorded at day 5.0 (1.9–8.0) (Figure 3A). Alternatively, censoring patients according to the PMA at transfusion, the median TFS was recorded at 28.0 weeks (26.0–29.4) (Figure 3B). We also explored whether the occurrence of various complications or death could be associated with different TFS. We observed that TFS was significantly lower in neonates with documented infections in comparison with other infants (HR 0.58, 95% CI 0.38–0.88, *p* = 0.004) or in neonates with multiple documented infections in comparison with patients with no or one single infectious episode (HR 0.33, 95% CI 0.17–0.64, *p* < 0.001). In contrast, suspected infections not followed by pathogen identification did not modify the TFS. Moreover, reduced TFS was significantly associated with the occurrence of IVH (HR 0.58, 95% CI 0.39–0.87, *p* = 0.004) or death (HR 0.36, 95% CI 0.21–0.62, *p* < 0.000), while no differences were observed in patients with and without NEC (Figure 4). Overall, these results suggest that concurrent infections, IVH, or death is associated with RBC need in an early phase of life.

**Table 1:** Clinical and transfusion characteristics of 100 neonates included in the study.

Gestational age at birth, weeks	27.0 (25.0–28.3)
Male gender	51 (51)
Twin	21 (21)
Birth weight, grams	795.0 (666.3–1,067.5)
Birth weight percentile	52.3 (17.9–69.0)
Htc at birth, %	45.5 (40.3–50.0)
Apgar score ^1m^	5 (4–7)
Apgar score ^5m^	8 (7–8)
Patent ductus arteriosus^§^	68 (68)
Intraventricular hemorrhage	53 (53.0)
Documented infections	46 (46.0)
Repeated documented infections	25 (25.0)
Suspected infections	47 (47.0)
Bronchopulmonary dysplasia^*^	24 (33.3)
Invasive ventilation, days	5.0 (0.0–13.5)
Non-invasive ventilation, days	22.5 (4.3–51.0)
Oxygen supplementation, days	17.0 (3.0–61.0)
ROP (all stages)^*^	48 (66.6)
Stage I ROP^*^	1 (1.3)
Stage II ROP^*^	22 (30.5)
Stage III or higher ROP^*^	25 (35.1)
Anti-VEGF therapy^*^	17 (23.6)
Deaths	33 (33)
Number of RBC units	3 (1–6)
Postnatal day at first RBC unit	5.5 (2.0–15.0)
Postmenstrual age at first RBC unit	28.0 (26.0–29.9)
Two or more RBC units	56 (56.0)
PLT transfusion	23 (23)
FFP transfusion	8 (8)

*Data are expressed as n (%) for categorical variables and median (IQR) for continuous variables*.

*Htc, central venous hematocrit; RBC, red blood cell; PLT, platelets; FFP, fresh frozen plasma; VEGF, vascular endothelial growth factor; ROP, retinopathy of prematurity; IQR, interquartile range*.

§ *Patients with hemodynamically significant patent ductus arteriosus are included*.

* *Data on retinopathy and bronchopulmonary dysplasia are related to 72 evaluable neonates; ROP stage refers to the maximal stage observed*.

### Transfusion-Free Survival in Neonates With Severe Retinopathy of Prematurity

**Figure 5:**
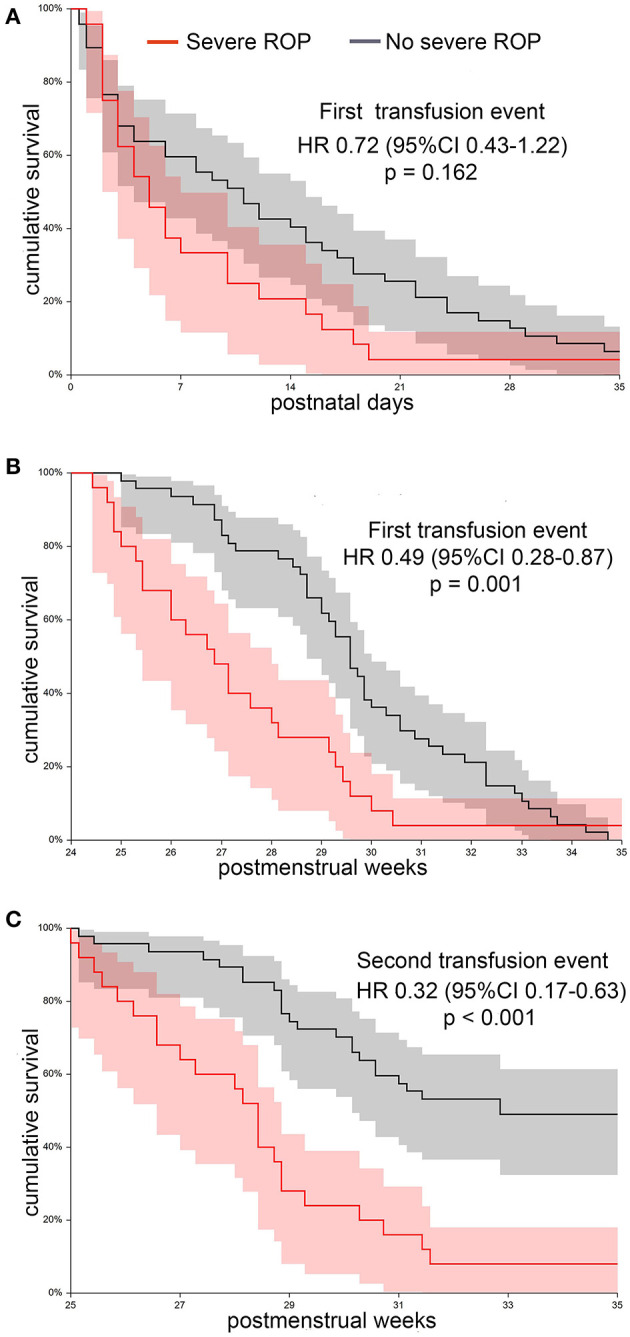
Cumulative transfusion-free survival in a cohort of 72 transfused neonates, grouped according to the occurrence of stage 3 or higher retinopathy of prematurity (ROP). **(A)** Transfusion-free survival estimated according to postnatal day at first RBC transfusion does not significantly differ between patients with and without severe ROP. **(B)** Transfusion-free survival estimated according to postmenstrual age at first transfusion is significantly lower in neonates developing severe ROP. **(C)** The difference between groups is even greater if survival to the second RBC transfusion event is considered. RBC, red blood cell.

All 100 neonates were subjected to periodical ROP assessment, according to the GA at birth. In total, 72 neonates survived until the PMA of 36 weeks and were considered eligible for estimating the cumulative TFS according to the occurrence of ROP. Table 2 shows clinical differences between neonates with and without severe ROP (i.e., stage III or higher) at univariate analysis. As expected, patients with severe ROP had lower GA, birth weight, and Apgar scores. Moreover, they had longer exposure to oxygen supplementation, experienced longer invasive and/or non-invasive ventilation, and more frequently developed BPD. In reference to other comorbidities, both groups had a similar incidence of documented infections, IVH, and surgical NEC. Neonates with severe ROP received a greater number of RBC transfusions. Figure 4 shows the cumulative TFS in patients with and without severe ROP. Neonates in the severe ROP group displayed a non-significant lower TFS calculated according to postnatal day (*p* = 0.162, Figure 5A). This difference was much more evident and statistically significant when TFS was calculated according to the PMA of recipients (*p* = 0.001, Figure 5B). In fact, neonates with severe ROP received transfusions at a significantly lower age than others (HR 0.49, 95% CI 0.28–0.87). At the PMA of 30 weeks, only 8% (95% CI 0–18) of neonates in the severe ROP group were transfusion-free, in comparison with 38% (95% CI 22–50) of neonates without severe ROP (Figure 5B). Twenty-four (96%) neonates with severe ROP and 32 (68%) without severe ROP were given more than one RBC transfusion (*p* = 0.007). Figure 5C illustrates shows that neonates in the severe ROP group received the second RBC unit at a significantly younger age than others (*p* < 0.001).

**Table 2:** Univariate analysis of clinical and transfusion data in neonates with and without severe ROP.

	**Severe ROP group** ***n* = 25**	**No severe ROP group** ***n* = 47**	***p*-value**
Gestational age at birth	25.9 (24.6–27.5)	28.0 (27.0–29.0)	<0.001
Male gender	12 (33.3)	24 (66.7)	1.000
Twin	7 (43.8)	9 (56.3)	0.392
Birth weight, g	710 (615–937)	980 (735–1,210)	0.003
Birth weight percentile	55.8 (7.4–72.1)	52.0 (19.0–69.8)	0.953
SGA	6 (24.0)	11 (23.4)	1.000
Hct at birth, %	48.0 (41.0–50.0)	45.0 (42.0–50.0)	0.593
Apgar score ^1m^	5 (4–6)	6 (5–7)	0.007
Apgar score ^5m^	7 (6.−8)	8 (8–9)	0.020
Patent ductus arteriosus^*^	19 (76.0)	27 (57.4	0.133
Intraventricular hemorrhage	13 (52.0)	21 (44.7)	0.624
Documented infections	13 (52.0	21 (44.7)	0.624
Surgical necrotizing enterocolitis	4 (16.0)	2 (42.5)	0.173
Bronchopulmonary dysplasia	13 (54.2)	11 (45.8)	0.019
Invasive ventilation, days	12.0 (5.0–25.3)	1.0 (0.0–6.0)	<0.001
Non-invasive ventilation, days	45.0 (31.5–88.0)	29.0 (16.0–52.0)	0.044
Oxygen supplementation, days	62.0 (32.0–95.5)	13.0 (1.0–6.01)	0.002
RBC units	6 (3–10)	2 (1–4)	<0.001
Postnatal day at first RBC unit	5 (2–11)	11 (3–22)	0.098
PMA at first RBC unit	26.9 (25.4–29.2)	29.6 (28.4–31.4)	<0.001
Two or more RBC units	24 (96.0)	32 (68.1)	0.007
PMA at second RBC unit	28.3 (26.2–29.2)	30.2 (28.7–34.6)	0.002
PLT transfusion	10 (41.7)	13 (58.3)	0.302
FFP transfusion	5 (55.6)	3 (44.4)	0.116

*Data are expressed as n (%) for categorical variables and median (IQR) for continuous variables*.

*Htc, central venous hematocrit values; RBC, red blood cell; PLT, platelets; FFP, fresh frozen plasma; ROP, retinopathy of prematurity; SGA, small for gestational age; PMA, postmenstrual age; IQR, interquartile range*.

* *Patients with hemodynamically significant patent ductus arteriosus are indicated*.

### Effect of Postmenstrual Age at Transfusion on Severe Retinopathy of Prematurity

Since we observed that severe ROP patients received transfusions earlier than others, we used ROC curve analysis to identify the PMA cutoff at which transfusion events were maximally associated with the risk for severe ROP. Considering that repeated transfusions are principally alleged to favor ROP, we performed the analysis regarding either the first transfusion event (analysis carried out on 72 patients) or the second transfusion event (analysis carried out on 56 patients receiving two or more transfusions). We found that receiving an RBC unit before 28 weeks of PMA predicted severe ROP with a sensitivity of 64% and a specificity of 78%. Moreover, receiving a second RBC unit before 29 weeks of PMA predicted severe ROP with a sensitivity of 75% and a specificity of 69%. Accordingly, we categorized all patients according to their PMA at first (< or ≥28 weeks) or second (< or ≥29 weeks) RBC transfusion event to investigate the association with the risk of severe ROP. Overall, neonates receiving the first RBC transfusion at PMA < 28 weeks had an OR of 6.57 (95% CI 2.24–19.26, *p* < 0.001). The risk was even higher for neonates receiving the second RBC transfusion at PMA < 29 weeks (OR 8.41, 95% CI 2.79–25.37, *p* < 0.001). We then evaluated the risk for ROP in multivariate logistic regression analysis including among covariates those variables that significantly differed between patients with and without severe ROP (Table 3). In addition to variables reflecting the severity of clinical conditions at birth (GA, BW, and Apgar scores), we included among covariates of the first model the principal acknowledged risk factors for ROP (duration of mechanical ventilation and oxygen supplementation) and the transfusion burden (number of RBC transfusions). The estimates of the abovementioned parameters were calculated at the time of diagnosis of severe ROP. In this model, GA at birth (OR 0.476, 95% CI 0.320–0.708, *p* < 0.001) and Apgar score at 5 min (OR 0.572, 95% CI 0.362–0.904, *p* = 0.017) significantly predicted severe ROP, with *p* = 0.920 at the Hosmer and Lemeshow test (Table 3). Then, we designed a second model, more focused on assessing the effect of TFS. We replaced GA at birth and the number of RBC transfusions by the age of the neonate at first (< or ≥28 postmenstrual weeks) or second (< or ≥29 postmenstrual weeks) RBC transfusion. We observed that PMA at second transfusion (OR 4.554 (95% CI 1.332–15.573, *p* = 0.016) and Apgar score at 5 min (OR 0.609, 95% CI 0.385–0.964, *p* = 0.034) outperformed other variables in predicting severe ROP (*p* = 0.802 at the Hosmer and Lemeshow test).

**Table 3:** Results of logistic regression analysis evaluating the impact of different factors on the risk for severe ROP in two different models.

	**OR**	**95% CI**	***p***
**Model 1**			
Gestational age at birth	0.476	0.320–0.708	<0.001
Apgar score ^5m^	0.572	0.362–0.904	0.017
**Model 2**			
2nd RBC transfusion at PMA <29 weeks	4.554	1.332–15.573	0.016
Apgar score ^5m^	0.609	0.385–0.964	0.034

*Model 1 incorporated as continuous covariates patient characteristics at birth (gestational age, birth weight, and Apgar scores at 1 and 5 min) and duration of invasive and non-invasive ventilation, oxygen supplementation, and number of RBC units, all estimated at the time of severe ROP diagnosis. In Model 2, the impact of transfusion-free survival was specifically investigated, by replacing gestational age at birth and number of RBC units by the postmenstrual age at first (<28 weeks) or second (<29 weeks) transfusion event. Only RBC transfusions were considered to estimate the transfusion-free survival*.

*ROP, retinopathy of prematurity; RBC, red blood cell; PMA, postmenstrual age*.

## Discussion

The association between repeated transfusions and ROP is supported by robust scientific evidence (7–9, 16, 25–28). The risk for developing ROP seems to be higher if RBC transfusions are administered at an early period of life (27, 29–31). In this regard, the findings emerging in the present study highlight two main points. The first one, regarding the period of transfusion, is that the risk for severe ROP conveyed by blood products likely depends on the age of recipients. This parameter can be expressed by the TFS measured according to the PMA and not postnatal days. In fact, while both parameters are informative regarding the transfusion timing, they are not equivalent in capturing different odds that transfusion events might have in promoting ROP. The second point is that PMA at the second transfusion may be even more informative in terms of severe ROP risk than PMA at the first transfusion. Indeed, PMA at second transfusion recapitulates the relevant role played by low GA and repeated transfusions in favoring more severe forms of ROP.

Even though the link between RBC transfusions and ROP is well-established, the influence of age at transfusion on the risk of developing the disease has been scarcely explored. In a recent study including preterm infants born ≤ 32 weeks, Lust et al. retrospectively evaluated the incidence of treated ROP in a series of 602 neonates receiving early transfusions (i.e., within the first 10 postnatal days) (27). A total of 1,034 neonates non-transfused (76% of controls) or transfused after the first 10 days of life (24%) served as a control group. The authors found that 91% of neonates with treated ROP were early transfused, with an OR of 3.8 (95% CI 1.8–8.1) after adjusting for GA and BW (27). The study included neonates with a higher GA than that in our analysis, and despite the large series of patients, it did not provide information about the PMA at which patients were transfused. In this regard, it is known that retinal development, and in particular retinal vascularization, occurs in specified weeks of gestation, which are therefore critical for ROP rise and progression. Indeed, it may be plausible that transfusions given at different PMAs might have a different damaging effect. Moreover, 76% of controls in the study of Lust et al. did not receive transfusions (27). In contrast, in order to correlate the risk for ROP to the age at which RBC units were given, we deemed it reasonable to exclude those neonates who did not receive transfusions.

A conceivable link between RBC transfusions and ROP might be represented by the consequent increase of adult hemoglobin (HbA) at the expense of fetal hemoglobin (HbF). It is widely acknowledged that even a moderate increase of HbF levels in patients with sickle cell disease or β thalassemia can modify the disease's clinical course (32, 33). Basically, functional differences between HbF and HbA molecules go far beyond the higher oxygen affinity and include extraordinary molecular stability of HbF, preventing the release of toxic-free heme groups (34). HbF also displays a higher pseudo peroxidase activity with significantly faster reconversion of the reactive ferryl-heme in comparison with HbA (35, 36), as well as a greater ability to generate unbound nitric oxide via the oxidative denytrosylation (37). The ensemble of all these characteristics explain the preventive effect of HbF from vaso-occlusive crisis in sickle cell disease, as well its vital importance for the progressive adaptation to the postnatal oxygen-rich environment in preterm neonates (12).

Recent studies have investigated the association between reduced HbF levels and the development of ROP. Stutchfield et al. demonstrated in a prospective cohort of 42 preterm neonates that infants with ROP had significantly lower HbF levels during their inpatient stay than those who did not develop ROP (61.75 vs. 91.9%, *p* = 0.0001) (29). Nevertheless, the temporal relationship between HbF decrease and development of ROP was not explored. In the subsequent prospective PacIFiHER trial, Jiramongkolchai et al. investigated HbF levels at weeks 31 and 34 of PMA of 60 neonates born at <31 weeks of GA (30). The authors demonstrated that low HbF values were associated with increased risk for mild or severe ROP (30). However, only three neonates with severe ROP were included in this study; no information related to transfusions was provided. Finally, Hellström et al. retrospectively evaluated HbF data collected at birth and in the first seven postnatal days in 385 infants (31). The authors found that in comparison with neonates without ROP, the 104 neonates developing any stage of ROP had equivalent HbF at birth but lower HbF levels in the first postnatal week. The probability for developing any ROP was about 60% in neonates with a mean HbF value of 40% in the first week of life, compared with only a 10% probability in those with a mean HbF value of 90% (31). Considering that the GA of enrolled patients was 26.4 ± 1.7 weeks, these findings strongly suggest that low-HbF-related causal mechanisms of ROP operate at a PMA lower than 31 weeks, in contrast to what Lust et al. suggested (27).

The protective effect from ROP exerted by HbF conceivably involves mechanisms other than the high oxygen affinity. Notably, data gathered in the PacIFiHER study clearly demonstrated that lower levels of HbF in preterm infants with ROP were associated with poorer indices of systemic oxygenation, as measured by median levels of SpO_2_ and PCO_2_ up to 34 weeks PMA (38). Our group has been working for years on an alternative transfusion approach for preterm neonates, based on RBC concentrates obtained from cord blood solidary donations at our public cord blood bank (39). We have carried out a recent “proof of concept” study demonstrating that cord RBC units prevent HbF depletion in transfused neonates (20). Likewise, we gathered evidence that neonates receiving transfusions from adult donors experienced at each transfusion a HbF reduction of about 40% of starting levels and that these values slowly recovered during the subsequent weeks if no additional transfusions were administered (20). On the contrary, the HbF decrease becomes exponential and irreversible in patients who were repeatedly transfused (20). Overall, these data might provide an explanation of our current observations, highlighting the importance of the timing of a second transfusion event. Unfortunately, there is no definite evidence about which HbF level is protective from ROP, as well as whether this threshold varies depending on different PMA, retinal development stages, and concomitant morbidities. This issue could be clarified by the ongoing study randomizing neonates born at <28 weeks to receive adult donor or cord blood RBC transfusions (NCT05100212). In the meantime, however, the findings emerging at the present analysis indicate that administering two or more consecutive RBC transfusions before 29 weeks of PMA may significantly amplify the risk for severe ROP.

Overall, the main limitation of this study resides in its retrospective design. The impact of data such as the intra-uterine growth restriction or chorion amnionitis was not investigated. Moreover, the study was conducted on a patient population accrued at a single institution, so our findings need to be validated in patients from different centers. Nevertheless, at variance with other studies, we included in the analysis only patients receiving transfusions and with GA at birth conveying a reasonable risk for severe ROP development. Finally, the study investigated the occurrence of severe ROP and not all stages of ROP: importantly, among various ROP stages, severe ROP has a foremost clinical impact, frequently requiring proper treatment or even evolving to blindness (2).

## Conclusions

Low GA remains the main risk factor for severe ROP: RBC transfusions may further increase this risk, but they are the unavoidable mainstay to treat anemia in preterm neonates. In this study, we show that comorbidities complicating the course of preterm birth, such as infections or IVH, can result in a shorter TFS. Considering that repeated RBC transfusions produce a critical decrease of HbF, it is conceivable that neonates receiving more than one unit before the PMA of 29 weeks may be exposed to a greater disturbance of retinal vascularization. Any strategy aimed at preventing this critical HbF decrease at this low age might potentially reduce the risk for severe ROP.

## Data Availability Statement

The raw data supporting the conclusions of this article will be made available by the authors, without undue reservation.

## Ethics Statement

The studies involving human participants were reviewed and approved by Fondazione Policlinico A Gemelli IRCCS Università Cattolica del Sacro Cuore, Rome. Written informed consent from the participants' legal guardian/next of kin was not required to participate in this study in accordance with the national legislation and the institutional requirements.

## Author Contributions

LT and PP designed the study, analyzed the data, and wrote the manuscript. AM, MBa, LP, CP, and NO collected and analyzed the clinical data. MBi and CV collected the transfusion data. BC, FS, and CG took care of the neonates. AB and DL performed the ophthalmological evaluations. GV critically reviewed the manuscript. All authors approved the submitted and final versions of the manuscript.

## Funding

The study was funded by Fondi di Ateneo, Progetti Linea D1 2019, Università Cattolica (Rome, Italy) to LT.

## Conflict of Interest

The authors declare that the research was conducted in the absence of any commercial or financial relationships that could be construed as a potential conflict of interest.

## Publisher's Note

All claims expressed in this article are solely those of the authors and do not necessarily represent those of their affiliated organizations, or those of the publisher, the editors and the reviewers. Any product that may be evaluated in this article, or claim that may be made by its manufacturer, is not guaranteed or endorsed by the publisher.
